# Comparison of hepatotoxicity of tegoprazan, a novel potassium-competitive acid blocker, with proton pump inhibitors using real-world data: A nationwide cohort study

**DOI:** 10.3389/fmed.2022.1076356

**Published:** 2023-01-11

**Authors:** Min-Gul Kim, Yong-Jin Im, Jong-Hwan Lee, Eun-Young Kim, Sang Woo Yeom, Jong Seung Kim

**Affiliations:** ^1^Department of Pharmacology, Medical School, Jeonbuk National University, Jeonju, South Korea; ^2^Center for Clinical Pharmacology, Jeonbuk National University Hospital, Jeonju, South Korea; ^3^Biomedical Research Institute of Jeonbuk National University Hospital, Jeonju, South Korea; ^4^Research Institute of Clinical Medicine of Jeonbuk National University, Jeonju, South Korea; ^5^Nanum Space Co. Ltd., Jeonju, Jeonbuk, South Korea; ^6^Department of Otolaryngology-Head and Neck Surgery, Medical School, Jeonbuk National University, Jeonju, South Korea; ^7^Department of Medical Informatics, Medical School, Jeonbuk National University, Jeonju, South Korea

**Keywords:** P-CAB, dexlansoprazole, esomeprazole, lansoprazole, omeprazole, pantoprazole, rabeprazole, PPI

## Abstract

**Background:**

Proton pump inhibitors (PPIs) are acid suppressants that are frequently prescribed in many countries to reduce heartburn. A potassium-competitive acid blocker (P-CAB; tegoprazan) was launched relatively recently that also inhibits gastric acid secretion. This study aimed to compare the hepatotoxicity of the six existing PPIs with P-CAB.

**Methods:**

This retrospective cohort study was conducted between January 2019 and December 2020 and included data from the total population of 50 million inhabitants in Korea. Propensity score (PS) matching was performed using 10 variables, and the differences in hepatotoxicity between P-CAB and the six PPIs were compared in a similar distribution. The primary endpoint was hepatotoxicity which included toxic liver disease, hepatitis, hepatic failure, liver transplantation, and other liver diseases.

**Results:**

The risk ratios (RR) of tegoprazan vs. the six PPIs (dexlansoprazole, esomeprazole, lansoprazole, omeprazole, pantoprazole, and rabeprazole) were all significant [RR: 0.70 (95% CI: 0.69–0.72), 0.81 (95% CI: 0.79–0.83), 0.61 (95% CI: 0.59–0.63), 1.17 (95% CI: 1.13–1.20), 0.61 (95% CI: 0.59–0.62), and 0.73 (95% CI: 0.71–0.75), respectively]. The risk ratio of tegoprazan vs. the six existing PPIs was 0.73 (95% CI: 0.72–0.75). The hazard ratios (HRs) of hepatotoxicity of the six PPIs to tegoprazan showed significantly higher values apart from omeprazole (HR: dexlansoprazole, 1.13; esomeprazole, 1.04; lansoprazole, 1.25; omeprazole, 0.77; pantoprazole, 1.26; rabeprazole, 1.15, respectively, and the six existing PPIs, 1.10).

**Conclusion:**

Using a large-scale data cohort analysis consisting of 50 million Koreans, tegoprazan did not induce higher hepatotoxicity compared with the six conventional PPIs.

## Introduction

Proton pump inhibitors (PPIs) are drugs that are frequently used to treat the symptoms associated with excessive gastric acid secretion. PPI is a treatment of choice drug to treat erosive esophagitis, GERD, and peptic ulcer disease and is an essential drug to increase the susceptibility of antibiotics in *Helicobacter pylori* eradication therapy (1–3). PPI has extensive Food and Drug Administration-approved indications such as (1) healing of erosive esophagitis, (2) maintenance of healed erosive esophagitis, (3) treatment of GERD, (4) risk reduction for gastric ulcer associated with NSAIDs, (5) *H. pylori* eradication to reduce the risk of duodenal ulcer recurrence in combination with antibiotics, (6) hypersecretory conditions including Zollinger–Ellison syndrome, and (7) short-term and maintenance treatment for duodenal ulcer (3). They irreversibly act as a disulfide bond to H^+^/K^+^ ATPase resulting in a strong gastric acid inhibitory effect.

Despite a long history of use and relatively well-established safety profiles, PPIs still cannot completely fulfill the requirements necessary to control gastrointestinal (GI) problems. PPIs are inactive when taken and do not take effect until they are absorbed from the small intestine (4). To counter these limitations, potassium-competitive acid blockers (P-CABs) were developed in the early 1980s, and these bind reversibly to the K^+^ binding domain of H^+^/K^+^ ATPase to prevent gastric acid secretion. However, earlier P-CABs, such as SCH28080, were discontinued due to hepatotoxicity in the human study (5, 6). A phase 2 clinical trial of YH4808 for reflux esophagitis was discontinued due to hepatotoxicity (7). AZD0865, which was introduced to treat erosive and non-erosive reflux disease, had liver toxicity issues in a randomized comparative trial, and its development was discontinued (8, 9).

Currently, there are only three P-CABs (revaprazan, vonoprazan, and tegoprazan) that have overcome these problems and have been approved by some regulatory agencies (10). In South Korea, tegoprazan has been on the market since 2019. Although there were no significant side effects in phase 3 clinical trials of tegoprazan, the concerns over hepatotoxicity were not completely dispelled (11). Dyspepsia, chest discomfort, and headache are all reported as drug-related, treatment-emergent adverse events for tegoprazan, but the fact that it belongs to the P-CAB drug group does not completely eliminate the concern over hepatotoxicity that existed in early P-CABs.

Recently, many studies have been conducted using real-world data (RWD), including electronic health records and insurance claims data, for post-market drug safety monitoring. Since South Korea has a national insurance system, the Health Insurance Review and Assessment (HIRA) Service, it is possible to evaluate the rare adverse drug reactions using the HIRA database.

This study aimed to compare the risk of hepatotoxicity with tegoprazan vs. PPIs.

## Methods

### Data source

Data selected from the anonymized HIRA database included patient demographics and detailed data from diagnosis. Since 99% of patients in Korea are enrolled in health insurance, the HIRA database is a dataset that is representative of the entire population. The database contains demographic data such as age, sex, and income quintile of all patients, and also medical information such as date of treatment, whether or not surgery was performed, outpatient or admitted as an inpatient, course of treatment, treatment details, diagnosis, and type of surgery.

### Study design

This nationwide population-based retrospective cohort study compared the risk of hepatotoxicity from tegoprazan compared with PPIs.

### Study population

The definition of the experimental cohort was “tegoprazan prescription”. The definition of the comparable cohort was “any PPI prescription”. The observation period was defined as the time from the first prescription of tegoprazan or PPI to the last day of tegoprazan or PPI plus up to 14 days. The inclusion criteria were as follows: (1) any tegoprazan or PPI drug user; and (2) age over 18 years. There were two exclusion criteria: (1) no follow-up data available in the database after the first prescription of tegoprazan or PPI; and (2) the patient taking two or more drugs among tegoprazan and six PPIs during the observation period. In the case of consecutive prescription dates in one patient, this case was evaluated as one case, and if prescriptions were received 28 days after the date of an earlier prescription, each was evaluated as a separate case.

### Propensity score matching

In this study, 10 variables that may affect hepatotoxicity were defined as follows (12, 13). Demography: (1) age (per 10-year increase; reference age was set as the average age of all patients); (2) sex (reference: male); (3) antibiotic usage, and (4) seven liver-related diagnostic records <1 year before PPI or P-CAB prescription (viral hepatitis, any type of malignancy, alcoholic hepatitis, chronic hepatitis, chronic hepatic failure, liver cirrhosis, and non-alcoholic fatty liver). To rule out hepatotoxicity from combined drugs, we used the combined antibiotics usage as matching variables in the propensity score matching. The antibiotics used are listed in Supplementary Table S1.

The independent variables in the comparable cohort had a distribution as similar as possible to the independent variables in the experimental cohort, and this was an essential part of the scientific method. For this purpose, 1:1 propensity score matching was performed six times (for P-CAB vs. the six PPIs) in the entire population. The propensity score was defined through logistic regression with 10 variables, and it was matched using the nearest greedy method. The potential risk of hepatotoxicity in the P-CAB and the PPI groups was equalized through propensity score matching.

### Outcomes

The primary outcome was hepatotoxicity. Overall hepatotoxicity was subdivided on the basis of hepatotoxicity-related outcomes: (1) toxic liver disease; International Classification of Diseases, 10th revision (ICD-10), code K71; (2) hepatic failure, code K72; (3) hepatitis, codes K75.2, K75.9; (4) liver transplantation, code Z94.4; and (5) other liver diseases, codes K76, K76.2, K76.7, and K76.9. Any hepatotoxicity was defined as a new entry of an ICD-10 code for hepatotoxic disease during the observation period.

### Statistical analysis

Data analysis was conducted between January 2019 and December 2020. The Cox proportional hazard model was used considering the prescription day of tegoprazan or PPIs and the observation period. The hazard ratio (HR), risk ratio (RR), and cumulative incidence rate were calculated using R 3.5.3 software (R Foundation for Statistical Computing, Vienna, Austria).

## Results

### Baseline characteristics and incidence rates for hepatotoxicity with the six PPIs or tegoprazan

**Figure 1:**
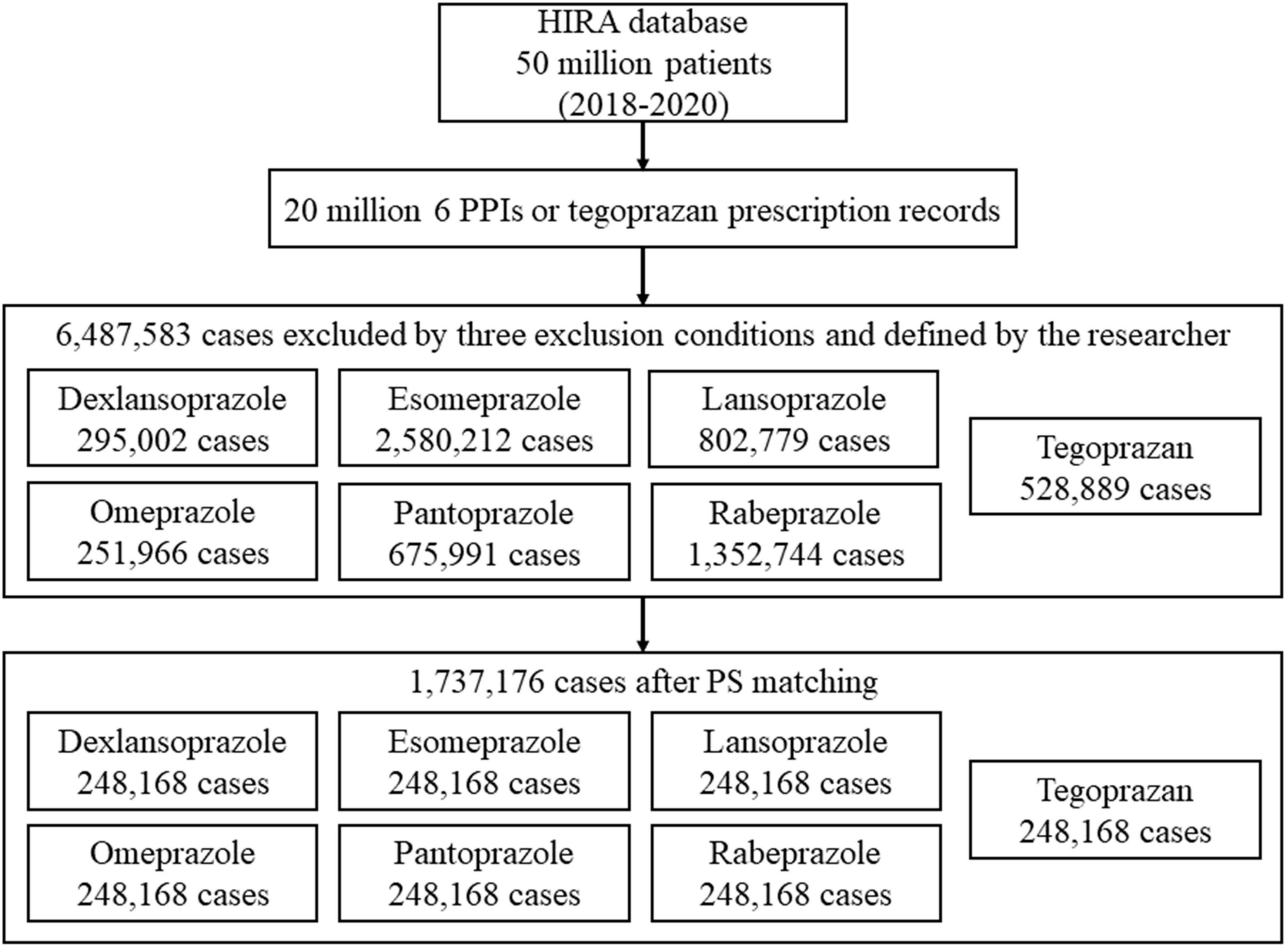
Flow chart for this study showing the study design.

Among the 50 million people included in the HIRA database, 6,487,583 cases were defined in the study population (dexlansoprazole, 295,002; esomeprazole, 2,580,212; lansoprazole, 802,779; omeprazole, 251,966; pantoprazole, 675,991; rabeprazole, 1,352,744; and tegoprazan, 528,889) (Figure 1).

Considering the 10 variables, 1:1 propensity score matching was performed, and 1,737,176 out of 6,487,583 cases were selected as data to be used for analysis.

The standardized mean difference (SMD) values for the six PPI groups and the tegoprazan group for age, sex, use of antibiotics, and the seven liver-related underlying diseases were all <0.2, indicating a homogeneous distribution (Table 1). In South Korea, only one tablet per day is recognized in health insurance claims for all PPIs and P-CAB; therefore, all doses are the same with one tablet per day. The duration of prescription with regard to the six PPIs and P-CAB was not different, and the SMDs were <0.2; thus, it can be seen that there was relatively no significant difference in the dose and duration of prescription for P-CAB and the six PPIs (14, 15).

**Table 1:** Standardized mean difference (SMD) values for the six PPI groups and the tegoprazan group for age, sex, use of antibiotics, and the seven liver-related underlying diseases were all <0.1, indicating a homogeneous distribution.

	**Tegoprazan**	**Dexlansoprazole**	**Esomeprazole**	**Lansoprazole**	**Omeprazole**	**Pantoprazole**	**Rabeprazole**	**SMD**
***N*** **(%)**	**248,168**	**248,168**	**248,168**	**248,168**	**248,168**	**248,168**	**248,168**	
Age, years [mean (SD)]	52.96 (16.55)	56.14 (16.73)	55.19 (16.23)	56.86 (16.86)	53.55 (16.70)	56.81 (16.78)	56.11 (16.25)	0.09
Sex female	134,092 (54.03)	130,116 (52.43)	130,157 (52.45)	126,928 (51.15)	128,774 (51.89)	125,366 (50.52)	132,949 (53.57)	0.03
Sex male	114,076 (45.97)	118,052 (47.57)	118,011 (47.55)	121,240 (48.85)	119,394 (48.11)	122,802 (49.48)	115,219 (46.43)	
Antibiotic	150,376 (60.59)	160,523 (64.68)	157,546 (63.48)	162,199 (65.36)	145,699 (58.71)	157,028 (63.27)	156,247 (62.96)	0.06
Viral hepatitis	7,590 (3.06)	9,244 (3.72)	8,939 (3.60)	10,078 (4.06)	7,530 (3.03)	10,859 (4.38)	8,832 (3.56)	0.03
Malignancy	17,233 (6.94)	29,371 (11.84)	21,622 (8.71)	31,620 (12.74)	18,453 (7.44)	30,466 (12.28)	19,626 (7.91)	0.08
Alcoholic hepatitis	3,015 (1.21)	3,589 (1.45)	3,808 (1.53)	4,800 (1.93)	3,004 (1.21)	4,712 (1.90)	3,894 (1.57)	0.03
Chronic hepatitis	6,102 (2.46)	6,362 (2.56)	7,432 (2.99)	8,411 (3.39)	6,910 (2.78)	8,584 (3.46)	6,899 (2.78)	0.03
Chronic hepatic failure	27 (0.01)	38 (0.02)	61 (0.02)	75 (0.03)	41 (0.02)	97 (0.04)	42 (0.02)	0.09
Liver cirrhosis	652 (0.26)	916 (0.37)	1,066 (0.43)	1,169 (0.47)	637 (0.26)	1,396 (0.56)	1,048 (0.42)	0.02
Non-alcoholic fatty liver	13,439 (5.42)	12,670 (5.11)	13,728 (5.53)	13,251 (5.34)	13,771 (5.55)	13,402 (5.40)	15,014 (6.05)	0.01
Duration, days [mean (SD)]	32.21 (63.00)	67.37 (135.21)	53.25 (113.77)	68.26 (140.74)	45.42 (102.21)	68.53 (143.66)	58.40 (125.89)	0.14
Hepatotoxicity	9,169 (3.69)	13,011 (5.24)	11,364 (4.58)	15,034 (6.06)	7,866 (3.17)	15,103 (6.09)	12,570 (5.07)	0.06
Toxic liver disease	987 (0.40)	1,741 (0.70)	1,298 (0.52)	2,215 (0.89)	978 (0.39)	1,945 (0.78)	1,262 (0.51)	
Hepatic failure	11 (0.00)	21 (0.01)	52 (0.02)	54 (0.02)	21 (0.01)	55 (0.02)	22 (0.01)	
Acute hepatitis	2,285 (0.92)	3,311 (1.33)	2,558 (1.03)	2,941 (1.19)	1,609 (0.65)	3,127 (1.26)	2,966 (1.20)	
Liver transplantation	36 (0.01)	70 (0.03)	285 (0.11)	136 (0.05)	79 (0.03)	645 (0.26)	59 (0.02)	
Other liver disease	5,850 (2.36)	7,867 (3.17)	7,170 (2.89)	9,688 (3.90)	5,178 (2.09)	9,330 (3.76)	8,261 (3.33)	

### Cumulative incidence rates of the six PPIs and tegoprazan

**Figure 2:**
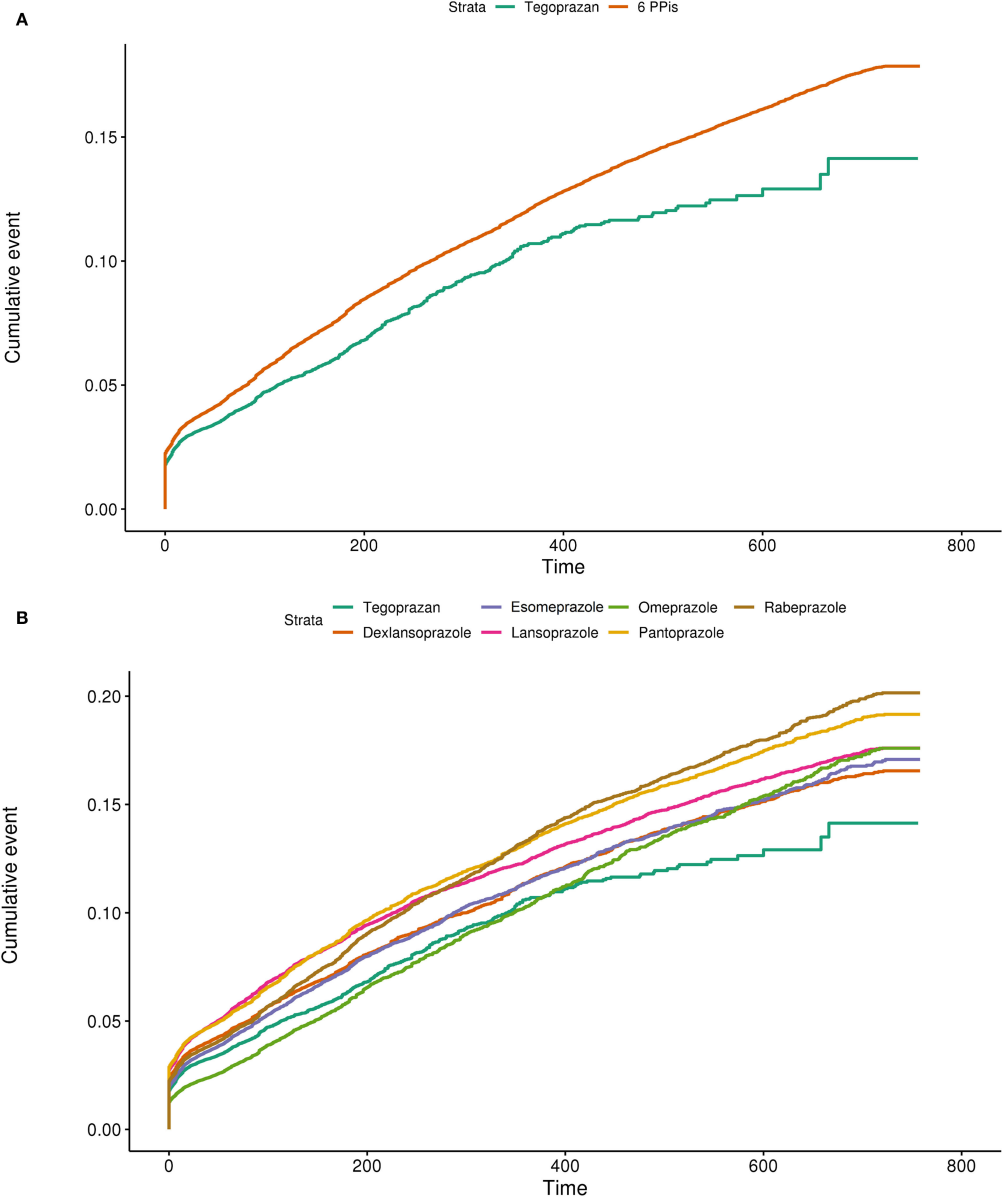
**(A)** Cumulative incidence plot using the Kaplan–Meier model comparing the hepatotoxicity of tegoprazan with the average of the six available PPIs. **(B)** Cumulative incidence plot using the Kaplan–Meier model comparing the hepatotoxicity of tegoprazan with dexlansoprazole, esomeprazole, lansoprazole, omeprazole, pantoprazole, and rabeprazole.

Tegoprazan had a lower cumulative incidence rate for overall hepatotoxicity than the six PPIs (Figure 2A). Subgroup analysis showed that tegoprazan clearly had a lower cumulative incidence rate than five of the PPIs (dexlansoprazole, esomeprazole, lansoprazole, pantoprazole, and rabeprazole) (Figure 2B).

### Risk ratios for hepatotoxicity of the six PPIs and tegoprazan

**Figure 3:**
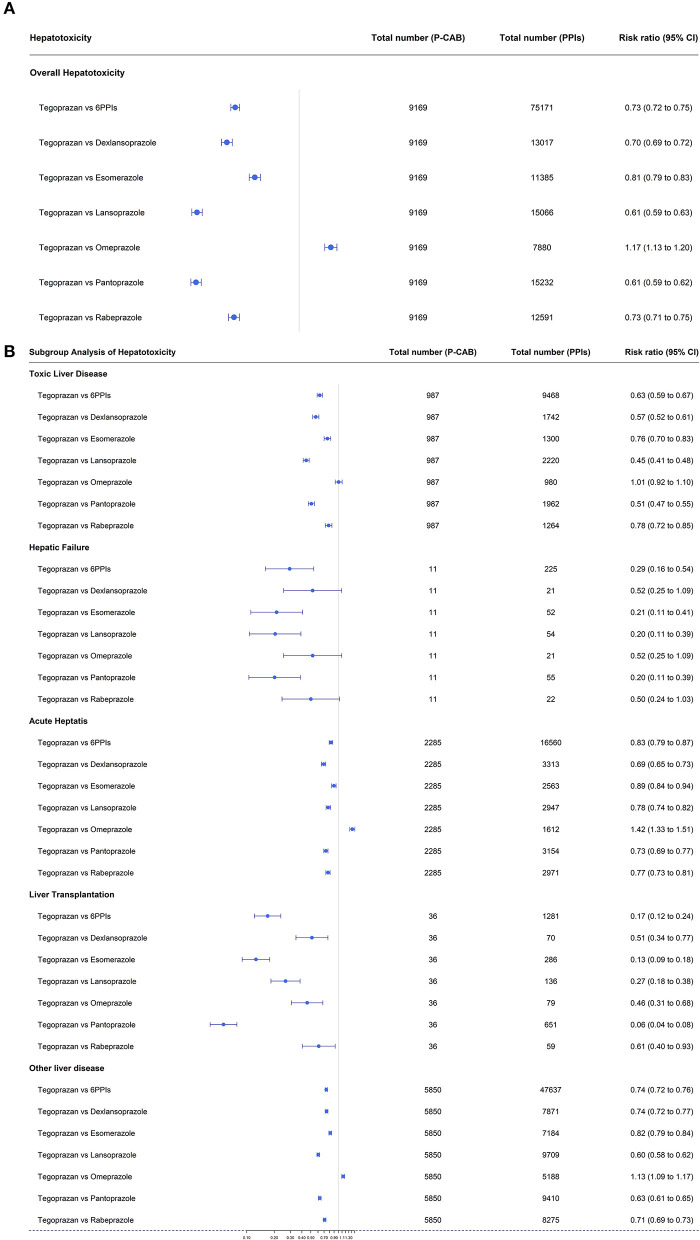
**(A)** Forest plots of overall hepatotoxicities showing the risk ratios for tegoprazan vs. the six available PPIs. **(B)** Forest plots of each hepatotoxicity in subgroup analysis showing the risk ratios for tegoprazan vs. the six available PPIs.

Tegoprazan had the lowest hepatotoxicity compared to the other PPIs. The risk ratio for tegoprazan vs. the six available PPIs was 0.73 (95% CI: 0.72–0.75) (Figure 3A). The risk ratios for tegoprazan vs. dexlansoprazole, esomeprazole, lansoprazole, omeprazole, pantoprazole, and rabeprazole were all significant [RR: 0.70 (95% CI: 0.69–0.72), 0.81 (95% CI: 0.79–0.83), 0.61 (95% CI: 0.59–0.63), 1.17 (95% CI: 1.13–1.20), 0.61 (95% CI: 0.59–0.62), and 0.73 (95% CI: 0.71–0.75), respectively] (Figure 3A).

In subgroup analysis, tegoprazan had a better result for RR [0.46 (95% CI: 0.31–0.68)] than omeprazole for liver transplantation, equivalent results for toxic liver disease and hepatic failure [RR; 1.01 (95% CI: 0.92–1.10), 0.52 (95% CI: 0.25–1.09), respectively], and a poorer outcome for acute hepatitis [RR; 1.42 (95% CI: 1.33–1.51)] (Figure 3B).

In all other subcategories, tegoprazan had lower RR values than the other five PPIs (Figure 3B).

### Cox proportional hazard analysis for hepatotoxicity of the six PPIs and tegoprazan

**Figure 4:**
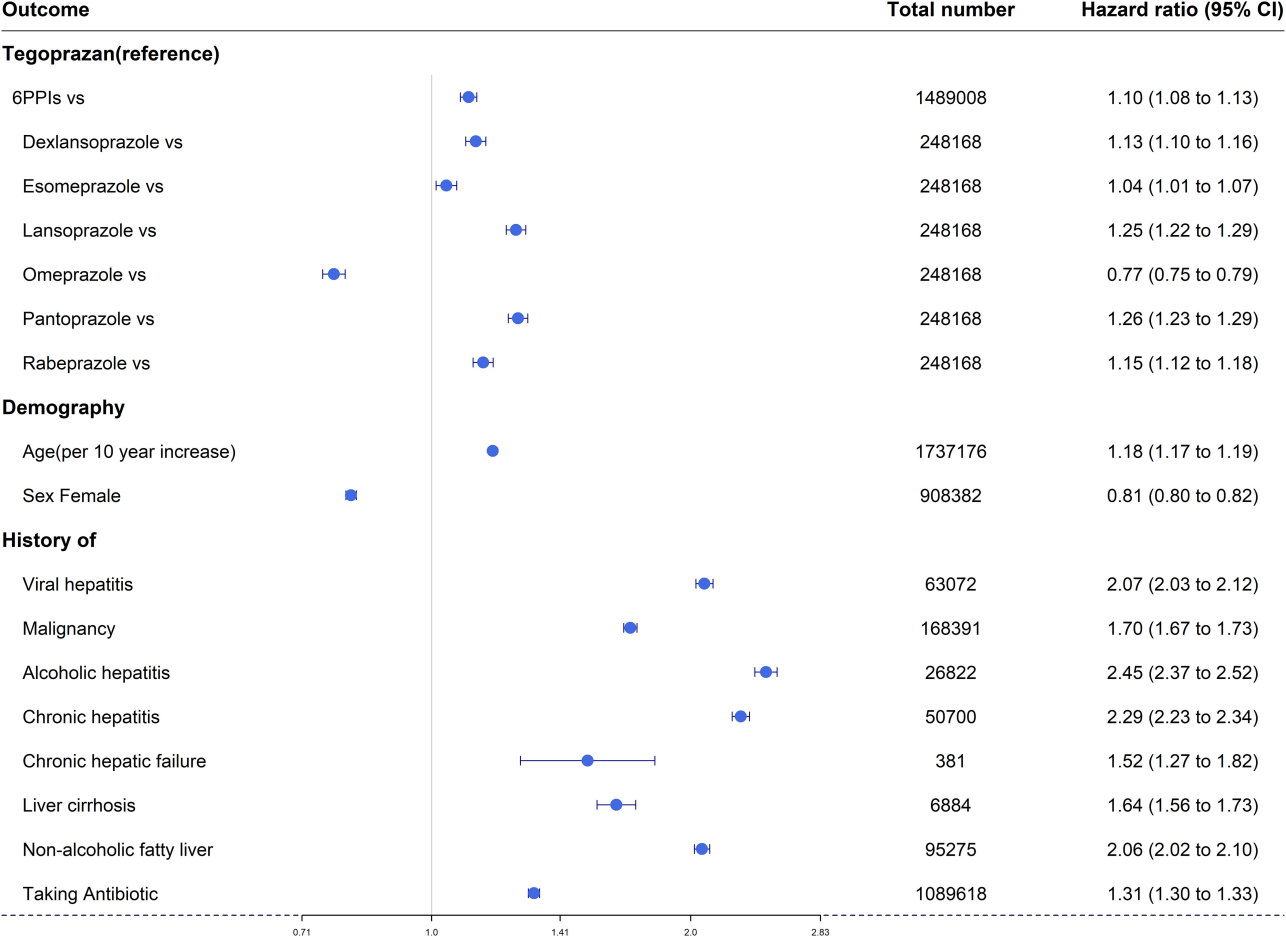
Forest plots of overall hepatotoxicities show the hazard ratio for the six available PPIs vs. tegoprazan.

A Cox proportional hazard model was used to consider the interrelationships of all variables associated with hepatotoxicity over time. Apart from omeprazole, all PPIs had higher adjusted HRs for overall hepatotoxicity compared with tegoprazan [HR: 6 PPIs, 1.10 (95% CI: 1.08–1.13); dexlansoprazole, 1.13 (95% CI: 1.10–1.16); esomeprazole, 1.04 (95% CI: 1.01–1.07); lansoprazole, 1.25 (95% CI: 1.22–1.29); omeprazole, 0.77 (95% CI: 0.75–0.79); pantoprazole, 1.26 (95% CI: 1.23–1.29); and rabeprazole, 1.15 (95% CI: 1.12–1.18)] (Figure 4).

Overall, hepatotoxicity increased with age [HR 1.18 (95% CI: 1.17–1.19)], and there was a lower risk in women [HR 0.81 (95% CI: 0.80–0.82)].

Prior antibiotic use was a factor that increased the risk of hepatotoxicity [HR 1.31 (95% CI: 1.30–1.33)], and all seven liver-related underlying diseases (viral hepatitis, any type of malignancy, alcoholic hepatitis, chronic hepatitis, chronic hepatic failure, liver cirrhosis, and non-alcoholic fatty liver) were significant factors that increased hepatotoxicity [HR: 2.07 (95% CI: 2.03–2.12), 1.70 (95% CI: 1.67–1.73), 2.45 (95% CI: 2.37–2.52), 2.29 (95% CI: 2.23–2.34), 1.52 (95% CI: 1.27–1.82), 1.64 (95% CI: 1.56–1.73), 2.06 (95% CI: 2.02–2.10), respectively] (Figure 4).

## Discussion

Tegoprazan, a novel P-CAB that has recently been introduced, provides a good alternative for gastric acid-related problems (14). It is an acid-stable, rapid-acting, long-lasting, and reversible inhibitor of H^+^-K^+^ ATPase (7). We have shown that the hepatotoxicity of tegoprazan is significantly lower than the six available PPIs.

Proton pump inhibitors are known to be relatively safe since they have been used for peptic ulcer disease and *H. pylori* eradication for the past 30 years (15). However, there are reports that they affect calcium metabolism during long-term use and cause an increased risk of hip fractures (16), and there are reports that they are associated with community-acquired pneumonia (17). In addition, since all PPIs are metabolized by CYP2C19, the cytochrome P450 isozyme, there are reports stating that caution should be exercised when co-administered with drugs such as clopidogrel (15). Theoretically, since PPIs are metabolized in the liver, they may be hepatotoxic, and this was reported in several articles (18–20).

Tegoprazan is a novel drug that was approved in 2018 and launched in March 2019 in Korea. We evaluated its hepatotoxicity by analyzing the claims data from HIRA for a 1-year period after launch (January 2019 to December 2020). In South Korea, National Health Insurance covers ~98% of the total Korean population; thus, large-scale longitudinal cohort data analysis was possible.

In this study, hepatotoxicity was analyzed by dividing the patients into five subgroups (toxic liver disease, hepatic failure, hepatitis, liver transplantation, and other liver diseases) based on the hepatotoxicity-related outcome. We analyzed hepatotoxicity using various statistical models—the incidence rate, the cumulative incidence rate using the Cox proportional hazard model, and the risk ratio.

This study had several limitations. First, there is an inherent disadvantage embodied in the research design of a retrospective study. Second, in the definition of hepatotoxicity, laboratory results or image tests were not included, and as it was a claim-based study, it was defined only by the ICD-10 code.

However, to overcome these disadvantages, we analyzed a large-scale dataset of ~250,000 individuals using propensity score matching, a technique that has only recently been introduced. It controlled tegoprazan and each PPI so that they had similar risks of hepatotoxicity. In addition, the strength of this study is that it identified the hepatotoxicity of PPI and P-CAB accurately by analyzing the entire population data of 50 million people in South Korea.

In conclusion, when analyzed using real data, tegoprazan did not induce higher hepatotoxicity compared to six conventional PPIs. Although the six existing PPIs are recognized as relatively safe drugs, in this study, tegoprazan was associated with lower hepatotoxicity than these PPIs. It reduced the risk of overall hepatotoxicity by ~27% compared with the PPIs. In the future, real-world data-based drug safety monitoring will be able to confirm the hepatotoxicity of tegoprazan more clearly.

## Data availability statement

The original contributions presented in the study are included in the article/Supplementary material, further inquiries can be directed to the corresponding author.

## Ethics statement

This study protocol was reviewed and approved by the Institutional Review Board of JBNUH (IRB number CUH 2020-04-029-002). As information used for analyses from the NHIS-National Sample Cohort database was encrypted, anonymized, and de-identified, informed consent was not required.

## Author contributions

M-GK: conceptualization, writing—review and editing, and project administration. Y-JI, J-HL, E-YK, and SY: data curation and statistical analysis. JK: data curation, analysis and interpretation of data, and writing—original draft preparation. All authors contributed to the article and approved the submitted version.
