# Supplementary material for: Comparison of hepatotoxicity of tegoprazan, a novel potassium-competitive acid blocker, with proton pump inhibitors using real-world data: A nationwide cohort study
Source: Front Med (Lausanne). 2023 Jan 11;9:1076356. doi: 10.3389/fmed.2022.1076356 (PMC9876560; doi:10.3389/fmed.2022.1076356)
Supplement: Supplementary file 1 [file Table_1.pdf]

Supplementary Table 1. Name and EDI code by ingredients of combined antibiotics usage as matching variables in the propensity score matching

NAME	EDI CODE BY INGREDIENTS
**ACETYLCYSTEINE GLYCINE THIAMPHENICOL**	101901AT, 102001AT
**ACETYLKITASAMYCIN**	102101AT
**ACETYLSPIRAMYCIN**	102102AT
**AMIKACIN SULFATE**	106802BI, 106803BI, 106804BI, 106805BI, 108101AC
**AMOXICILLIN**	108101AT, 108102AS, 108103AC, 108103AT, 108201BI, 111301BI
**AMOXICILLIN SODIUM (AS AMOXICILLIN)**	108202BI, 108203BI, 108601AC, 108601BI, 108602AS, 108603AC, 108603BI, 108604AS, 111101BI
**ASTROMYCIN SULFATE**	112701AC
**AZITHROMYCIN**	112701AT, 112702AS, 112801BI
**AZLOCILLIN SODIUM**	112802BI, 112803BI, 113001BI
**AZTREONAM**	113002BI, 113101AT
**BACAMPICILLIN HCL**	115501AT
**BACAMPICILLIN HYDROCHLORIDE**	113102AT
**BENZATHINE PENICILLIN G**	115502BI, 123401BI
**CARBENICILLIN SODIUM**	123402BI, 124901BI, 124902BI, 125201AC
**CEFACLOR**	125202AS, 125203AS, 125204AC, 125204AT, 125205AC, 125205AT, 125206AS, 125207AT, 125208AS, 125209AG, 125210AT, 125301AT
**CEFACLOR HYDRATE**	125201AG
**CEFADROXIL**	125302AC, 125303AS, 125304AC, 125305AS, 125306AS, 125401BI
**CEFAMANDOLE NAFATE**	125402BI, 125501BI
**CEFAMANDOLE SODIUM**	125502BI, 125601AC
**CEFATRIZINE PROPYLENE GLYCOL**	125602AS, 125701BI
**CEFAZOLIN SODIUM**	125702BI, 125703BI, 125704BI, 125801BI
**CEFBUPERAZONE SODIUM**	125802BI, 125901AC
**CEFDINIR**	125901AG, 125902AC, 126001AG
**CEFDITOREN PIVOXIL**	126001AT, 126101BI
**CEFEPIME HYDROCHLORIDE HYDRATE**	126102BI, 126103BI, 126201AT
**CEFETAMET PIVOXIL HCL**	126301AC
**CEFETAMET PIVOXIL HYDROCHLORIDE**	126202AP, 126202AT

**CEFIXIME**	126302AP, 126401BI
**CEFIXIME HYDRATE**	126302AC
**CEFMENOXIME HYDROCHLORIDE**	126403BI, 126501BI
**CEFMETAZOLE SODIUM**	126502BI, 126503BI, 126601BI
**CEFMINOX SODIUM**	126602BI, 126701BI
**CEFODIZIME SODIUM**	126702BI, 126801BI
**CEFONICID SODIUM**	126802BI, 126901BI
**CEFOPERAZONE SODIUM**	126902BI, 126903BI, 127001BI
**CEFORANIDE**	127002BI, 127101BI
**CEFOTAXIME SODIUM**	127102BI, 127103BI, 127104BI, 127201BI
**CEFOTETAN DISODIUM (AS CEFOTETAN)**	127202BI, 127203BI, 127301BI
**CEFOTIAM HEXETYL HCL**	127402AT, 127501BI
**CEFOTIAM HYDROCHLORIDE**	127302BI, 127303BI, 127401AT
**CEFOXITIN SODIUM**	127502BI, 127601BI
**CEFPIMIZOLE SODIUM**	127602BI, 127701BI, 127702BI, 127801BI
**CEFPIROME SULFATE**	127802BI, 127901AT
**CEFPODOXIME PROXETIL**	127902AS, 127903AS, 128001AT
**CEFPROZIL**	128003AS, 128101AC
**CEFPROZIL HYDRATE**	128002AS
**CEFRADINE**	130103AS, 130105AC, 130106AS, 131001AC
**CEFRADINE HYDRATE**	130101BI, 130105BI
**CEFROXADINE**	128201BI
**CEFSULODIN SODIUM**	128202BI, 128301BI
**CEFTAZIDIME HYDRATE**	128302BI, 128303BI, 128401AG
**CEFTERAM PIVOXIL**	128401AT, 128402AT, 128501BI
**CEFTEZOLE SODIUM**	128502BI, 128503BI, 128504BI, 128601AS,
**CEFTIBUTEN**	128602AC, 128603AC, 128604AC, 128701BI
**CEFTIZOXIME SODIUM**	128702BI, 128801BI
**CEFTRIAXONE SODIUM HYDRATE**	128802BI, 128803BI, 128804BI, 128805BI, 128901AT
**CEFUROXIME AXETIL (AS CEFUROXIME)**	128902AS, 128903AT, 128904AT, 129001BI
**CEFUROXIME SODIUM**	129002BI, 129003BI, 129301BI
**CEPHACETRILE SODIUM**	129302BI, 129401AC
**CEPHALEXIN**	129402AC, 129501BI
**CEPHALEXIN LYSINATE**	129502BI, 129503BI, 129601BI
**CEPHALEXIN SODIUM**	129701BI
**CEPHALOTHIN SODIUM**	129702BI, 129801BI, 129802BI, 129803BI, 129804BI, 130001BI
**CEPHAZEDONE SODIUM**	130002BI, 130003BI, 130101AC

**CEPHRADINE**	130102BI, 130104BI, 130107BI
**CHLORAMPHENICOL**	131003AC, 131101BI
**CHLORAMPHENICOL SODIUM SUCCINATE**	132601AC
**CICLACILLIN**	132602AS, 132603AC, 133901AC, 133902AC, 134101AT
**CIPROFLOXACIN HCL(AS CIPROFLOXACIN)**	134103AT
**CIPROFLOXACIN HYDROCHLORIDE**	134103AC, 134105AT, 134108AT
**CIPROFLOXACIN HYDROCHLORIDE (AS CIPROFLOXACIN)**	134101BI, 134104BI, 134106BI, 134109AT, 134901AT
**CLARITHROMYCIN**	134902BI, 134903AS, 134903AT, 134904AT, 134905AS, 135401AC
**CLINDAMYCIN HYDROCHLORIDE**	135402AC, 135501AS
**CLINDAMYCIN PALMITATE HCL**	135601BI
**CLINDAMYCIN PHOSPHATE**	135603BI, 135605BI, 137901BI
**COLIMYCIN**	137902BI, 137903BI, 138001AC
**COLISTIN SODIUM METHANESULFONATE**	141001AC
**DEMECLOCYCLINE HCL**	143001BI
**DIBEKACIN SULFATE**	143002BI, 147301AT
**DIRITHROMYCIN**	149501AC
**DOXYCYCLINE GUAIACOLSULFONATE**	149701AC
**DOXYCYCLINE HYCLATE HYDRATE**	149701AT, 149701BI
**DOXYCYCLINE HYDRATE**	149501AT, 149601AC, 149702AS, 149703AC, 152001AT
**ENOXACIN**	152002AT, 153501AC
**ERYTHROMYCIN ESTOLATE**	153501AT, 153502AS, 153601AG
**ERYTHROMYCIN PROPIONATE**	153601AT, 153602AG, 153602AT, 153701AT
**ERYTHROMYCIN STEARATE**	153801AT
**ERYTHROMYCIN STINOPRATE**	153901AC
**ERYTHROMYCIN(ENTERIC COATED)**	154001AC, 154001AT, 154002AC, 154003AC, 154101BI
**ERYTHROMYCINE LACTOBIONATE**	154102BI, 154103BI, 159401AT

**FLEROXACIN**	159402AT, 159501BI
**FLOMOXEF SODIUM**	159801AC
**FLUCLOXACILLIN SODIUM**	159802BI, 159803AC, 159803BI, 163201AC
**FOSFOMYCIN CALCIUM**	163201AT, 163202AT, 163203AC, 163203AT, 163204AS, 163301BI
**FOSFOMYCIN SODIUM**	163302BI, 163303BI, 163304BI, 163401AG
**FOSFOMYCIN TROMETAMOL**	163401AP, 163402AP, 164101AS
**FUSIDIC ACID**	165101BI
**GENTAMICIN SULFATE**	165103BI, 165105BI, 165106BI, 165109BI, 165110BI, 165111BI, 165112BI, 167401AT
**GREPAFLOXACIN**	177701BI
**ISEPAMICIN SULFATE**	177702BI, 177703BI, 179201AT
**JOSAMYCIN**	179401BI
**KANAMYCIN SULFATE**	181701AT
**LENAMPICILLIN HCL**	182401AC
**LEUCOMYCIN**	182401AT, 183203AT, 183205BI, 183206BI, 184201AC
**LEVOFLOXACIN HYDRATE**	183202AT, 183202BI, 183203BI
**LINCOMYCIN HYDROCHLORIDE**	184202BI, 184203AC, 184204BI, 184205BI, 184206BI, 184901AC
**LOMEFLOXACIN HYDROCHLORIDE**	184903AT, 184904AT, 185301AC
**LORACARBEF**	185302AS, 186401AT
**LYMECYCLINE**	188301BI
**MECILLINAM**	188302BI, 188303BI, 190701BI
**MEROPENEM**	190702BI, 190703BI, 190704BI, 191301AC
**METAMPICILLIN SODIUM**	191302AC, 191302AT, 191302BI, 191601AC
**METHACYCLINE HYDROCHLORIDE**	191602AC, 193101AC
**METHYLOL CEPHALEXIN LYSINATE**	193101AT, 193102BI, 193103BI, 194401BI
**MEZLOCILLIN SODIUM**	194402BI, 195101BI
**MICRONOMICIN SULFATE**	195103BI, 195401AC
**MIDECAMYCIN**	195501AS
**MIDECAMYCIN ACETATE**	195501AT, 195502AS, 195901AC
**MINOCYCLINE HCL**	195901BI, 195903AC, 197401BI
**MINOCYCLINE HYDROCHLORIDE**	195905AP
**MOXALACTAM SODIUM**	197402BI, 197403BI, 199001AT
**NALIDIXIC ACID**	200701BI

**NETILMICIN SULFATE**	200702BI, 200703BI, 200704BI, 203301AT
**NORFLOXACIN**	203302AC, 203302AT, 203303AC, 203303AT, 203901AT
**OFLOXACIN**	203902BI, 203904AT, 203907AT, 203907BI, 206701AC
**OXOLINIC ACID**	206701AT, 207401AC
**OXYTETRACYCLINE HYDROCHLORIDE**	207403AC, 209201BI
**PAROMOMYCIN SULFATE**	209501AT
**PEFLOXACIN METHANESULFONATE**	209501BI, 210001BI
**PENICILLIN G POTASSIUM CRYSTAL**	210002BI, 210101BI
**PENICILLIN G SODIUM CRYSTAL**	210102BI, 210201AT
**PENICILLIN V POTASSIUM**	213001AC
**PIPEMIDIC ACID TRIHYDRATE**	213001AT, 213002AC, 213003AC, 213003AT, 213101BI
**PIPERACILLIN SODIUM**	213102BI, 213103BI, 213104BI, 214301AT
**PIVAMPICILLIN**	214302AT, 214401AT
**PIVMECILLINAM HCL**	223801BI
**RIBOSTAMYCIN SULFATE**	223802BI, 224801AT
**ROKITAMYCIN**	225301AT
**ROXITHROMYCIN**	225302AG, 225302AT, 225303AT, 225304AS, 227901BI
**SISOMICIN SULFATE**	227902BI, 227903BI, 229101AT, 229103BI, 230701AT
**SPARFLOXACIN**	230702AT, 230801BI
**SPECTINOMYCIN HCL**	230901AT
**SPECTINOMYCIN HYDROCHLORIDE**	230802BI
**SPIRAMYCIN**	230902AS, 232101BI
**STREPTOMYCIN SULFATE**	232601BI
**SULBENICILLIN SODIUM**	232602BI, 232701BI
